# Long term results of accelerated 9 mW corneal crosslinking for early progressive keratoconus: the Siena Eye-Cross Study 2

**DOI:** 10.1186/s40662-021-00240-8

**Published:** 2021-05-01

**Authors:** Cosimo Mazzotta, Frederik Raiskup, Farhad Hafezi, Emilio A Torres-Netto, Ashraf Armia Balamoun, Giuseppe Giannaccare, Simone Alex Bagaglia

**Affiliations:** 1 Departmental Ophthalmology Unit, USL Toscana Sud-Est, Campostaggia, Siena, Italy; 2 Siena Crosslinking Center, 53035, Monteriggioni, Siena, Italy; 3 Department of Ophthalmology, C.G. Carus University Hospital, Dresden, Germany; 4 ELZA Institute, Dietikon, Switzerland; 5 Medical Faculty, University of Geneva, Geneva, Switzerland; 6 Roski Eye Institute, University of Southern California, Los Angeles, CA USA; 7 Department of Ophthalmology, University of Wenzhou, Wenzhou, China; 8 Department of Ophthalmology, Federal University of Sao Paulo, Sao Paulo, Brazil; 9 Ashraf Armia Eye Clinic and Al Watany Eye Hospital, Watany Research and Development Centre, Cairo, Egypt; 10 Department of Ophthalmology, University Magna Graecia of Catanzaro, Catanzaro, Italy

**Keywords:** Crosslinking, Keratoconus, Accelerated crosslinking, Epi-off, Dresden accelerated protocol, 9 mW crosslinking, CXL, ACXL

## Abstract

**Purpose:**

To assess clinical results of the 9 mW/5.4 J/cm^2^ accelerated crosslinking (ACXL) in the treatment of progressive keratoconus (KC) over a span of 5 years.

**Methods:**

The prospective open non-randomized interventional study (Siena Eye-Cross Study 2) included 156 eyes of 112 patients with early progressive KC undergoing the Epi-Off 9 mW/5.4 J/cm^2^ ACXL at the Siena Crosslinking Centre, Italy. The mean age was 18.05 ± 5.6 years. The 20-min treatments were performed using the New KXL I (Avedro, Waltham, USA), 10 min of 0.1% HPMC Riboflavin soaking (VibeX Rapid, Avedro, Waltham, USA) and 10 min of continuous-light UV-A irradiation. Uncorrected distance visual acuity (UDVA), corrected distance visual acuity (CDVA), Kmax, coma, minimum corneal thickness (MCT), surface asymmetry index (SAI), endothelial cell count (ECC) were measured, and corneal OCT performed.

**Results:**

UDVA and CDVA improved significantly at the 3rd (*P* = 0.028), Δ + 0.17 Snellen lines and 6th postoperative month, respectively (*P* < 0.001), Δ + 0.23 Snellen lines. Kmax improved at the 6th postoperative month (*P* = 0.03), Δ − 1.49 diopters from the baseline value. Also, coma aberration value improved significantly (*P* = 0.004). A mild temporary haze was recorded in 14.77% of patients without affecting visual acuity and without persistent complications. Corneal OCT revealed a mean demarcation line depth at 332.6 ± 33.6 μm.

**Conclusion:**

The 5-year results of Epi-Off 9 mW/5.4 J/cm^2^ ACXL demonstrated statistically significant improvements in UCVA and CDVA, corneal curvature and corneal higher-order aberrations which confers a long-term stability for progressive ectasia. Based on the results of the Siena Eye-Cross Study 2, the 9 mW/5.4 J/cm^2^ ACXL is a candidate to be  the natural evolution of Epi-Off CXL treatment for the management of early progressive corneal ectasia, and thus optimize clinic workflow.

## Background

Conventional riboflavin UV-A induced corneal crosslinking (CXL) with epithelium removal (Epi-Off) [1, 2] represents a cost-effective treatment [3] with documented long-term efficacy in stabilizing progressive keratoconus [4, 5] and secondary ectasia [6, 7] in randomized clinical trials [8] and open non-randomized studies both in young adult [9] and pediatric populations [10], which thus reduce the need of corneal transplants for keratoconus by 25% [11] to 50% [12].

The standard energy dose of 5.4 J/cm^2^ delivered in 30 min at 3 mW/cm^2^ UV-A irradiance after 30 min of isotonic 0.1% Riboflavin-Dextran 20% stromal soaking (the Dresden protocol) [13], was approved in the United States by the Food and Drug Administration (U.S. FDA) in April 2016 [14], and requires a long treatment time of 1 h [15]. In order to shorten the duration of CXL, improve patients’ comfort and reduce wound-related stimuli of the corneal stroma, different Epi-Off accelerated crosslinking protocols (ACXL) [16–26] were tested based on the equal-dose principle stated in the Bunsen Roscoe’s law of reciprocity [27] and on preclinical lab studies [28] addressing the biomechanical equivalence between conventional Epi-Off CXL and ACXL with continuous and pulsed UV-light exposure [29–31]. The most commonly used settings in Epi-Off ACXL treatments were the 9 mW/5.4 J/cm^2^ × 10 min of continuous UV-light exposure [17], the 18 mW/5.4 J/cm^2^ × 5 min^22^, the 15 mW/5.4 J/cm^2^ × 12 min with pulsed-UV-light exposure [25] and the 30 mW/5.4 J/cm^2^ × 3 min continuous [23] and pulsed-light [16]. Richoz et al. [32] demonstrated that the Bunsen-Roscoes’ law does not apply in full for ACXL because the biomechanical effect of CXL decreases significantly when using high-UV irradiance with short irradiation times due to the reduced stromal oxygen diffusion capacity which may be a limiting factor that reduces overall treatment efficiency. Clinical studies [33–35] demonstrated that the 9 mW/5.4 J/cm^2^ ACXL gained comparable visual outcomes with conventional 3 mW/cm^2^ Dresden protocol with keratoconus stabilization [36, 37].

We report the long-term (5-years) clinical results of the 9 mW/5.4 J/cm^2^ ACXL protocol also named “Dresden Accelerated Protocol” in a large cohort of patients performed in Italy at the Siena Crosslinking Centre named “Siena Eye-Cross Study 2”.

## Patients and methods

### Dataset and study design

The prospective long-term open non-randomized, non-comparative interventional Siena Eye-Cross Study 2 was approved by the institutional review board (IRB) of the Siena Crosslinking Center following the tenets of the Declaration of Helsinki and included 156 eyes of 112 patients who underwent an Epi-Off 9 mW/5.4 J/cm^2^ ACXL procedure for progressive KC and completed the 5-year follow-up. All patients were affected by progressive (stage I and II) KC and were enrolled in the ACXL treatment protocol from January 2014 to April 2015. The prospective open non-randomized interventional Siena Eye-Cross Study 2 evaluating the 9mW Dresden accelerated CXL protocol included all patients who completed the 5-year follow-up. Bilateral treatments included 88 of 156 eyes (56.4%) that showed a preoperative bilateral KC progression. All patients 18 years old and under (*N* = 20), including 40 eyes, belong to bilateral treatments (25.6%). Patients 18 years old and younger represent 45.5% of the total number of bilateral treatments. The remaining 24 patients who underwent a bilateral treatment, including 48 eyes (54.5%), were between 19 and 25 years old. The unilateral treatments were performed in 68 of the 156 eyes (43.6%) and included 19 eyes of 19 patients with unilateral Keratoconus (12.1%), while the remaining unilateral ACXL treatments included 49 eyes of 49 patients ranging from 26 to 31 years old, showing no progression in the fellow eye during the follow-up. Bilateral treatments were performed after a minimum time interval of 30 days to a maximum of 60 days (mean 40 days). Seventy patients were male (87.5%). The mean age at the time of enrolment in the treatment protocol was 18.05 ± 5.6 years (range: 8–31 years).

### Inclusion criteria

Progressive stage II KC was identified according to Krumeich’s staging system [38]. Progression of KC was defined as an increase in apical keratometry (AK) ≥ 1 diopter (D) on the anterior corneal topography using a Scheimpflug-Placido corneal tomography system Sirius, Costruzione Strumenti Oftalmici (C.S.O.), Florence, Italy; minimum corneal thickness (MCT) reduction ≥ 10 μm; worsening of uncorrected distance visual acuity (UDVA) and corrected distance visual acuity (CDVA) ≥ 0.1 decimal equivalent or change of ≥ 0.5 in mean refractive spherical equivalent (MRSE) in the last 6 months of clinical and instrumental observation [39]. MCT was at least 400 μm (epithelium included), measured with no-contact optical pachymetry. Corneas were clear with no sub-apical opacities or scars and no markedly visible Vogt’s striae, no previous infectious keratitis or autoimmune diseases and no severe dry eye. All patients included in the study gave their specific written informed consent.

### Surgical procedure

All treatments were performed at the Siena Crosslinking Centre, Italy, by the same surgeon (CM). The 9 mW/5.4 J/cm^2^ ACXL treatment was performed using the KXL I system (Avedro, Waltham, MS, USA) under topical anesthesia (4% Oxibuprocaine chlorydrate 1.6 mg/0.4 mL drops) that was applied 10 min before the treatment after a premedication with 2% pilocarpine instilled 30 min before the operation. After applying a closed valves eyelid speculum, a 9-mm diameter epithelium was removed with a blunt metal spatula. After epithelial removal, a dextran-free plus hydroxyl-propyl methylcellulose (HPMC) disposable 0.1% riboflavin isotonic solution (VibeX Rapid, Avedro, Waltham, MA, USA) was instilled for one drop every minute for 10 min of corneal soaking, before starting continuous light UV-A irradiation. During UV-A-irradiation, 2 drops of riboflavin solution were administered every 2.5 min for a total of 10 min of UV-A exposure at 9 mW/cm^2^ of UV-A power and standard Fluence of 5.4 J/cm^2^. At the end of the UV-A irradiation, the cornea was washed with balanced saline solution (BSS) and medicated with preservative-free netilmicin plus dexamethasone, cyclopentolate eyedrops and dressed with a therapeutic bandage soft contact lens for 4 days. After therapeutic contact lens removal, fluorometholone 0.2% eye-drops (tapered 3 times/day) and sodium hyaluronate 0.2% lacrimal substitutes were administered for 6 to 8 weeks.

### Measurements and devices

Ophthalmic evaluations were performed before CXL and at all follow-up visits (1, 3, 6, 12, 24, 36, 48 and 60 months). The evaluation included UDVA, CDVA and a slit-lamp clinical examination. Scheimpflug based corneal tomography (Sirius, CSO, Florence, Italy) was used to measure maximum curvature simulated k reading (Kmax), coma high-order aberration, MCT, tomography derived SAI and topographic cylinder (CYL). Anterior segment optical coherence tomography (AS-OCT) with the I-Vue (Optovue, Freemont CA, USA) was performed to assess the demarcation line depth at the first post-operative month. Endothelial cell count was measured by the I-Konan Non-Co Robot (Konan Inc., Hyogo, Japan) preoperatively and every year until the end of the study.

### Statistical analysis

A two-tailed paired samples *t* test was used to compare each baseline measurement with the respective follow-up measurements. Differences with *P* < 0.05 were considered statistically significant. Data were collected and analyzed with PRISM 6.0 GraphPad Software (La Jolla, California, USA).

## Results

### Baseline data

Visual acuity was represented in Snellen lines and decimal equivalents (d.e.). UDVA was 0.37 ± 0.11 and mean CDVA was 0.63 ± 0.09. Mean steepest corneal curvature (Kmax) was 55.70 ± 3.10 D. MCT was 443.76 ± 37.17 μm. Vertical coma high-order aberrations value was 1.01 ± 0.016 μm. SAI values were 4.77 ± 2.43. Mean CYL values were 2.94 ± 1.74 D. Endothelial cell count (ECC) was 2437.75 ± 287.6 cell/mm^2^. Baseline data are displayed in Table 1.

**Table 1:** Baseline characteristics of studied cohort

Baseline total characteristics 156 eyes of 112 patients 88 eyes, 44 patients bilateral ACXL 68 eyes, 68 patients unilateral ACXL	Value (mean)	SD or %
Mean age (years)	18.05	± 5.6
Male	70	85%
UDVA Snellen (d.e.)	0.37	± 0.11
CDVA Snellen (d.e.)	0.63	± 0.09
Kmax (D)	55.70	± 3.10
Minimum corneal thickness (μm)	443.76	± 37.17
Coma (μm)	1.01	± 0.016
SAI	4.77	± 2.43
CYL (D)	2.94	± 1.74
Endothelial cell (cell/mm^2^)	2437.75	± 287.6

*ACXL* = accelerated crosslinking; *SD* = standard deviation; *UDVA* = uncorrected distance visual acuity; *CDVA* = corrected distance visual acuity; *d.e*. = decimal equivalents; *SAI* = surface asymmetry index; *CYL* = topographic cylinder

**Fig. 1:**
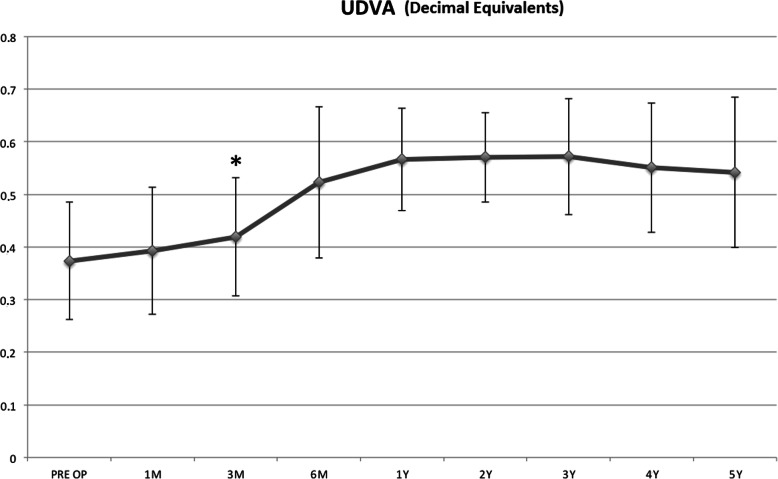
Uncorrected distance visual acuity (UDVA) improved from baseline, becoming statistically significant at the 3rd postoperative month (*P* = 0.028) and remained significant until the end of follow-up

UDVA improved from baseline, becoming statistically significant at the 3rd postoperative month (*P* = 0.028) and remained significant until the end of follow-up (Fig. 1).

**Fig. 2:**
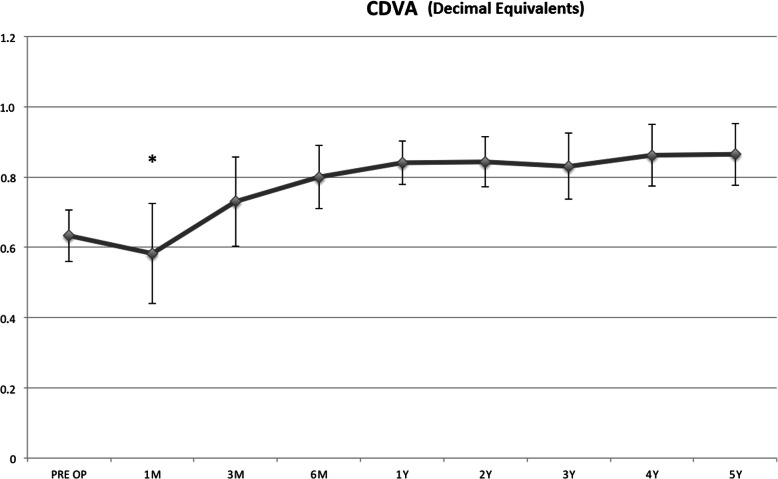
Corrected distance visual acuity (CDVA) showed a statistically significant improvement at the 1st (*P* <  0.001) postoperative month, remaining significant until the end of follow-up

CDVA showed a statistically significant improvement at the 3rd (*P* <  0.001) postoperative month, remaining significant until the end of follow-up, (Fig. 2).

**Fig. 3:**
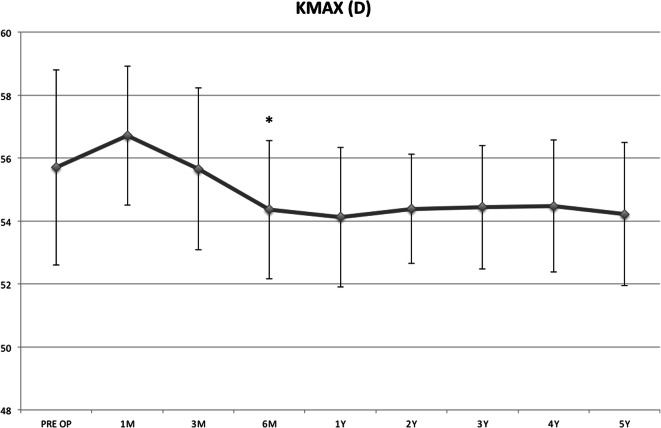
The baseline and follow-up measurements of Kmax showed that it improved significantly at 6th postoperative month, from 55.7 D to 54.36 D (*P* = 0.03) and remained statistically significant up to the 5th year of follow-up (*P* = 0.001) with a delta of − 1.49 D

The baseline and follow-up measurements of Kmax documented that the value improved significantly at the 6th postoperative month, from 55.7 D to 54.36 D (*P* = 0.03) and this improvement remained statistically significant up to the 5th year follow-up (*P* = 0.001; delta − 1.49 D, Fig. 3).

**Fig. 4:**
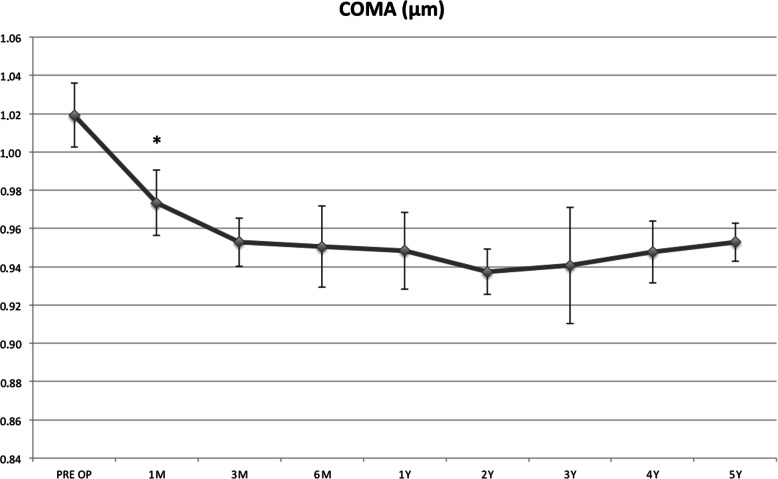
The coma high order aberration mean value improved during the follow-up, becoming statistically significant at the 1st postoperative month (*P* = 0.004)

The coma high order aberration mean values improved during the follow-up, becoming statistically significant at the 1st postoperative month (*P* = 0.004, Fig. 4).

**Fig. 5:**
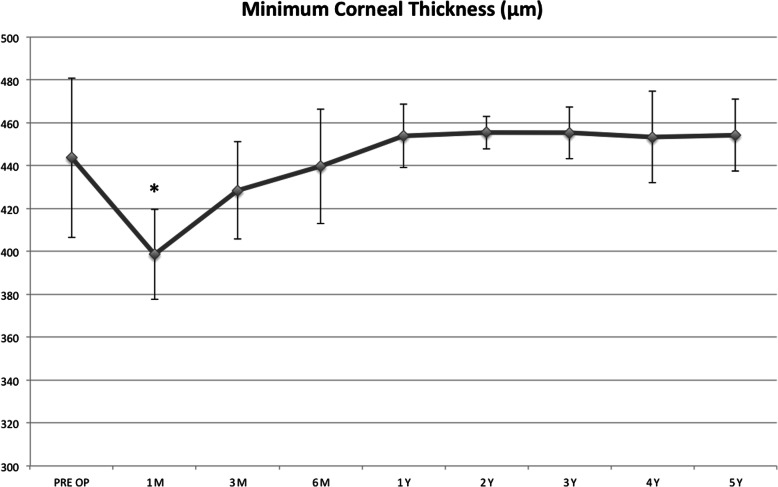
Tomographic mean minimum corneal thickness (MCT) value decreased significantly at the 1st (*P* = 0.0001) and 3rd postoperative month (*P* = 0.03), returning to baseline at the 6th month and maintaining then a substantial stability up to the end of the follow-up period

Tomographic mean MCT value decreased significantly at the 1st (*P* = 0.0001) and 3rd postoperative month (P = 0.03), returning to baseline at the 6th month and maintaining a substantial stability up to 5-years follow-up (Fig. 5).

**Fig. 6:**
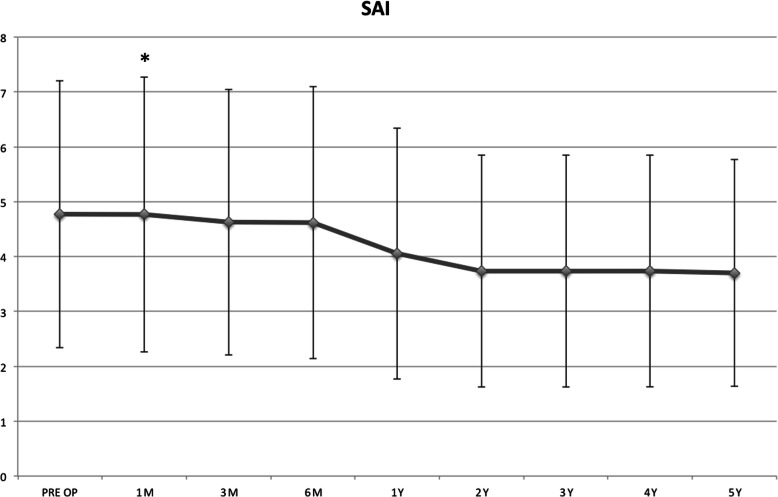
Topographic SAI showed a significant change at the 1st postoperative month (*P* = 0.04)

The topographic SAI values showed a significant change at the 1st month (*P* = 0.04), (Fig. 6).

**Fig. 7:**
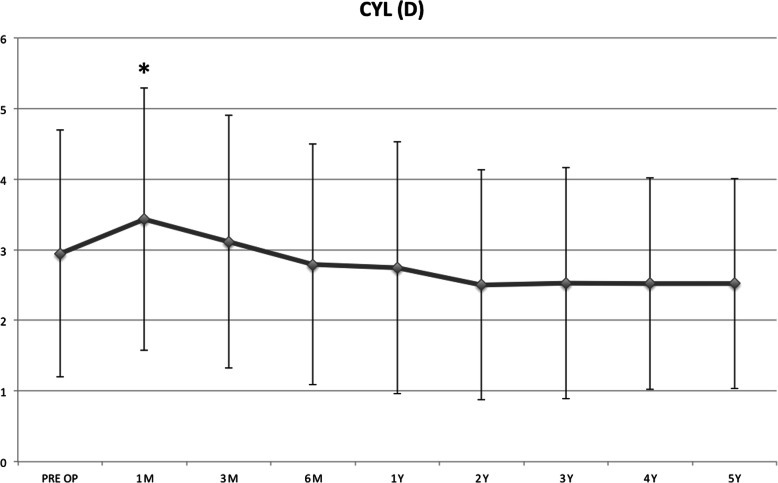
Mean topographic cylinder (CYL) values remained stable along the entire follow-up period despite a positive trend. In the pediatric group, the CYL values showed a temporary significant improvement at the 1st month (*P* = 0.034) but became insignificant after the 3rd postoperative month

The CYL average values remained stable along the entire follow-up in the whole study cohort, despite a positive trend. In the pediatric group, the CYL values showed a temporary significant improvement at the 1st month (*P* = 0.034) but became insignificant after the 3rd postoperative month (Fig. 7).

ECC showed no significant postoperative variations during any follow-up point (*P* > 0.05).

**Fig. 8:**
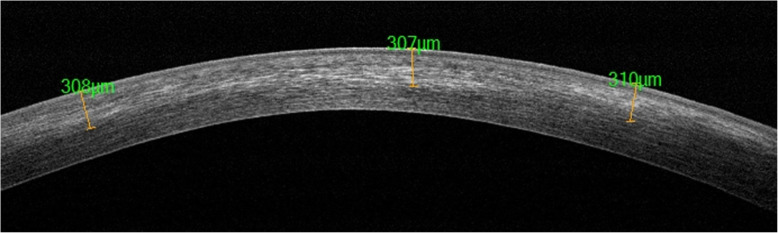
AS-OCT evaluation of the demarcation line depth at the 1st postoperative month showed a depth of 332.6 ± 23.6 μm

AS-OCT evaluation of the demarcation line depth at the 1st postoperative month showed a depth of 332.6 ± 23.6 μm in the overall study cohorts (Fig. 8).

The overall follow-up results are reported in Table 2.

**Table 2:** Overall study results

	UDVA Snellen (decimal equivalent)	*P*	CDVA Snellen (decimal equivalent)	*p*	Kmax (D)	*P*	Coma (D)	*P*	MCT (μm)	*P*	SAI	*P*	CYL (D)	*P*
Baseline	0.37		0.63		55.70		1.019		443.76		4.777		2.944	
Δ1m	0.02	0.23	05	0.052	1.01	0.052	−0.046	0.004*	−45.1	0.0001*	−0.005	0.045**	−0.48	0.34
Δ3m	0.04	0.028**	−0.05	0.013*	−0.05	0.067	−0.67	< 0.001*	− 15.2	0.0302**	0.143	0.834	−0.16	0.78
Δ6m	0.15	0.0001*	0.17	0.001*	−1.34	0.03*	−0.069	< 0.001*	−3.96	0.23	0.154	0.81	0.15	0.73
Δ1y	0.19	0.0001*	0.21	0.001*	−1.58	0.001*	−0.071	< 0.001*	10.17	0.817	0.716	0.232	0.21	0.65
Δ2y	0.20	0.001*	0.22	0.0002*	−1.33	0.001*	−0.082	< 0.001*	11.74	0.52	1.038	0.075	0.44	0.38
Δ3y	0.20	0.0001*	0.23	0.001*	−1.3	0.001*	−0.079	< 0.001*	11.67	0.56	1.039	0.0757	0.42	0.32
Δ4y	0.18	0.0001*	0.23	0.0067*	−1.23	0.001*	−0.072	< 0.001*	9.64	0.682	1.039	0.058	0.42	0.25
Δ5y	0.17	0.0001*	0.23	0.0031*	−1.49	0.001*	−0.067	< 0.001*	10.50	0.579	1.071	0.0503	0.418	0.25

**Indicates statistical significance (*P* > 0.05). * indicates statistical significance (*P* > 0.01). Δ change relative to the respective baseline value; *UDVA* = uncorrected distance visual acuity; *CDVA* = corrected distance visual acuity; *Kmax* = maximum keratometry; *MCT* = minimum corneal thickness; *SAI* = surface asymmetry index; *CYL*=topographic cylinder

### Adverse events

No postoperative infections, persistent haze or endothelial cell failure were encountered during the entire follow-up period in the whole study cohort. In 16 eyes (10.25%) of 13 patients, a mild temporary haze at the 1st postoperative month at slit lamp examination was observed. However, this did not affect visual acuity and disappeared between the 3rd and the 6th month visit once topical steroids therapy was started (preservative-free 1 mg/mL dexamethasone eye-drops, tapered 4 times a day for 4 to 8 weeks). Thirteen eyes (8.33%) of 8 patients had a Kmax progression of 1 D within the 2nd-3rd year follow-up visits, returning to baseline value after 30 ± 6 months. All patients with such progression were affected with severe allergic papillary conjunctivitis. No retreatment was performed in the entire 5-year follow-up period. Data for adverse events are reported in Table 3.

**Table 3:** Adverse events

Total adverse events	Eyes (n)	Total (%)	Patients (n)	Total (%)	Time of presentation
Temporary haze	16	10.25	13	11.6	1st month
K max progression > 1 D	13	8.33	8	5.1	24th–30th month
Corneal transplants	0	0	0	0	0
Overall progression	13	8.33	8	5.1	24th–30th month

## Discussion

**Fig. 9:**
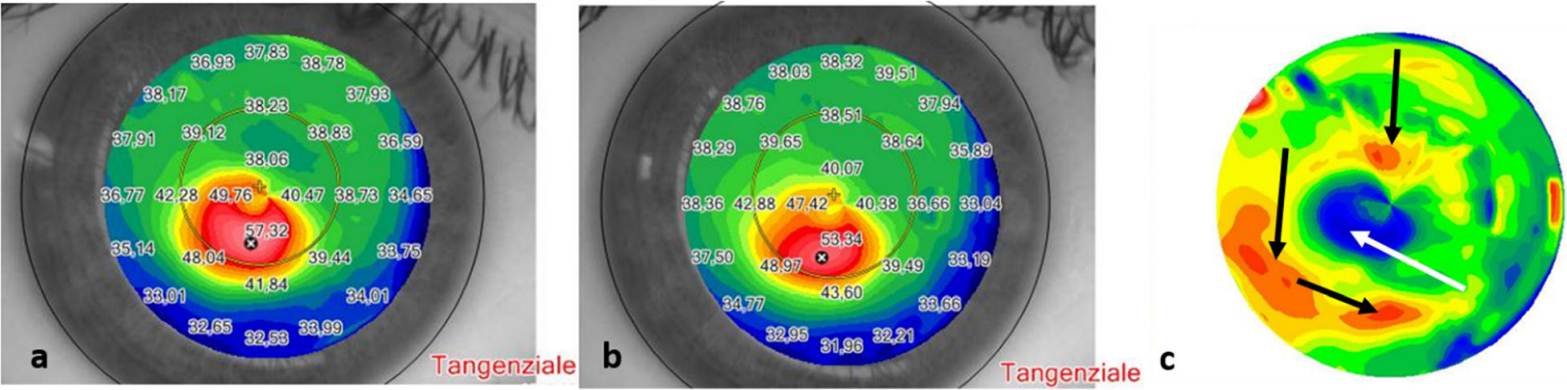
Topographic patterns after 9 mW/5.4 J/cm^2^ ACXL showing preoperative (**a**) and 5-year postoperative (**b**) corneal curvature reduction. The differential tangential map (**c**) shows the typical central flattening (white arrow) and the paracentral steepening (black arrows) surrounding the apex of KC

The 5-years follow-up results after accelerated epithelium-off CXL (9 mW/cm^2^ for 10 min) in the broadest case series of 156 KC eyes of 88 consecutive patients and with the longest follow-up currently reported internationally, showed clinically significant improvements in UDVA and CDVA, corneal curvature reduction with typical central flattening of the apical region of the KC with paracentral compensatory steepening of the flattest areas, inducing an improved corneal symmetry and corneal higher-order aberrations reduction (Fig. 9). In addition, the procedure was well tolerated in all patients, conferring a long-term (over 5-years) stability for progressive KC. No significant adverse events such as postoperative infections, persistent haze or endothelial cell failure were encountered during the entire follow-up period. A mild temporary haze that did not affect visual acuity was observed at the 1st postoperative month in around 15% of patients, disappearing after the 3rd month of clinical observation after topical steroid therapy.

Maximum corneal curvature progression of 1 D, which returned to baseline, was recorded between the 24th and 36th month follow-up in 13 eyes of 8 patients (8.33%). Patients that returned to baseline after initial functional improvement were all affected with allergies and were eye-rubbers. Interestingly, despite no retreatments being performed in the 5-years follow-up, none of these patients showed worsening in either UDVA or CDVA. Severe allergic conjunctivitis, eye-rubbing, and blepharitis-associated ocular surface chronic inflammation, however, were recognized as possible causes of poorer response and corneal topography instability [40].

The 5-years progression, intended as Kmax deterioration of 1 D or more occurred in 8.33% of patients, however, the improvement in CDVA did remain significant. AS-OCT evaluation of the demarcation line depth at the 1st postoperative month showed a depth of 332.6 ± 33.6 μm in the overall study cohort. To ensure a long-lasting stability of keratoconus and secondary ectasia, UV-A power settings and exposure time were targeted to allow a treatment penetration at least at 250 μm. Such depth of treatment penetration would allow a large portion of the anterior stroma to be cross-linked, which would be desirable especially since the anterior 40% of the central corneal stroma is the stiffest region of the cornea. Moreover, according to Kohlhaas *et al.* [41], the CXL treatment significantly stiffens the cornea only in the anterior 200 μm. The depth-dependent stiffening effect can be explained by the absorption behavior for UV-A on a riboflavin-soaked cornea, where about 70% of UVA irradiation is absorbed within the anterior 200 μm and only 20% in the next 200 μm [42]. This aspect may affect long-term ectasia stabilization according to different crosslinking penetration rate [43]. While maintaining a standard 5.4 J/cm^2^ dose delivery, it was demonstrated by Mazzotta* et al.* in the "*M nomogram*" for the standardized treatment of all thickness ectatic corneas including the thin and ultrathin KC maintaining the standardized fluence of the original Dresden protocol (5.4J/cm^2^) in all cases, that UV-A power can be calibrated between 9 and 15 mW/cm^2^ with continuous or pulsed-light mode of exposure [44]. Indeed, the stress-strain testing data from an experimental study performed by Krueger *et al.* [29] demonstrated a substantial biomechanical equivalence between 3 mW/5.4 J/cm^2^ CXL for 30 min of continuous UVA light exposure, 9 mW/5.4 J/cm^2^ ACXL for 10 min of continuous UVA light exposure and 15 mW/5.4 J/cm^2^ ACXL with continuous or pulsed-light exposure [25].

Despite laboratory data from Richoz *et al.* [32] showing lower stiffening effect due to oxygen availability in ACXL procedures, the 9 mW/5.4 J/cm^2^ was estimated to have the best oxygen diffusion profile in the ACXL procedures panorama. Several clinical studies also demonstrated that the 9 mW/5.4/cm^2^ Epi-Off ACXL obtained comparable visual outcomes and KC stabilization with conventional 3 mW/5.4 J/cm^2^ CXL (Dresden protocol). Moreover, the AS OCT and in vivo confocal microscopy (IVCM) studies by Mazzotta *et al.* [45] showed that the mean depth of the demarcation line after the 9 mW/5.4 J/cm^2^ ACXL was at 332 ± 20 μm, the nearest to the conventional 3 mW/5.4 J/cm^2^ CXL Dresden protocol 320 μm (range 270–350 μm) [1, 4].

The 9 mW/5.4 J/cm^2^ ACXL for 10 min of continuous light UVA exposure was first demonstrated to be effective in stabilizing topographic parameters after 12-months of follow-up in mild-moderate KC affected corneas by *Elbaz et al* [17]. An improvement in the UDVA and stabilization of all tested corneal parameters were noted after the treatment. Moreover, the 9 mW/5.4 J/cm^2^ ACXL was safe for corneal endothelium, stabilizing the progression of iatrogenic ectasia with a significant reduction in topographic keratometric values and a significant increase in CDVA, comparable with conventional 3 mW/cm^2^ CXL in a mid-term (two-years) follow-up as recently documented by *Turhan et al* [37]. A recent study performed by Sadoughi *et al.* [33] comparing the conventional 3 mW/5.4 J/cm^2^ CXL to 9 mW/5.4 J/cm^2^ ACXL in patients with bilateral progressive KC where the fellow eyes were randomly assigned to conventional 3 mW/5.4 J/cm^2^ CXL or to 9 mW/5.4 J/cm^2^ ACXL, revealed similar refractive, visual, keratometric and aberrometric outcomes after 12 months of follow-up. The study confirmed that the one and two-years functional outcomes of the 9 mW/5.4 J/cm^2^ ACXL protocol were similar and comparable with the conventional 3 mW/5.4 J/cm^2^ CXL.

In a comparative study of 29 eyes treated with 9 mW/cm^2^ for 10 min, Kirgiz A *et al.* [35] reported that ACXL using 10 min of UVA irradiance at 9 mW/cm^2^ showed better topographic and coma values improvements vs. 5 min of UVA at 18 mW/cm^2^ irradiance, independent of keratoconus severity. Furthermore, Lang *et al.* [34] in a comparative study using standard CXL protocol and accelerated protocols in patients with progressive keratoconus reported that Epi-Off 3 mW/5.4 J/cm^2^ CXL for 30 min, 5.4 J/cm^2^ and 9 mW/5.4 J/cm^2^ ACXL for 10 min showed similar improvements in Kmax and CDVA.

Kobashi *et al.* [36] in a recent meta-analysis compared the clinical results of ACXL and standard corneal collagen cross-linking (SCXL) in progressive keratoconus of randomized controlled trials, which showed a comparable efficacy and safety profile at the 1-year follow-up between the two procedures.

Besides performing stress-strain experiments, CXL efficacy has also been shown to drop from SCXL to short ACXL protocols using inflation tests or air-puff tonometry. SCXL and ACXL have shown similar results [46, 47].

Although 9 mW/5.4 J/cm^2^ ACXL for 10 min provides less biomechanical increase under laboratory conditions [32], it is not known exactly how much stiffening each keratoconic cornea would need for the progressive character of the disease to be stabilized.

Our study data suggest that 9 mW/cm^2^ for 10 min may be sufficient to efficiently prevent progression. Nevertheless, we are currently evaluating in the laboratory whether a high-fluence ACXL Epi-Off setting using 9 mW/7.2 J/cm^2^ for 13 min and 20 s would further increase the biomechanical stability. If this were the case, then an increased fluence might provide an additional level of efficacy to the treatment.

## Conclusion

The 5-years results of the 9 mW/cm^2^ Epi-Off ACXL with 5.4 J/cm^2^ energy, evaluated in the Siena Eye-Cross Study 2, demonstrated statistically significant improvements of UCVA, CDVA, corneal curvature and corneal higher-order aberrations, and thus confers a long-term stability for progressive KC. In our series, bilateral treatments were performed in 56.4% of patients and the second eye was operated after a minimum time interval of 30 days to a maximum of 60 days (mean 40 days). If keratoconus progression is documented in both eyes, the treatment of the second eye should be performed in a timely fashion, especially in pediatric patients to avoid further risk of progression and higher economic burden [48]. The 9 mW/5.4 J/cm^2^ for 10 min ACXL protocol – supported by photochemistry, microstructural and long-term clinical data – became our Epi-Off CXL treatment of choice, which reduces CXL treatment time from 1 h to 20 min allowing better patient comfort while maintaining overall CXL safety and efficacy. According to the Siena Eye-Cross Study 2, this accelerated crosslinking protocol is a candidate to be the natural evolution of the original Dresden Epi-Off CXL treatment for the management of early progressive corneal ectasia, thus optimizing clinic workflow, and patient compliance being really efficiacious, less time-consuming and more cost-effective.

## Data Availability

Not applicable.
